# Proton pump inhibitor use after kidney transplantation and graft outcomes: A matched national cohort study

**DOI:** 10.1097/MD.0000000000051046

**Published:** 2026-10-09

**Authors:** Ahmed E. Aly, Vineeth R. Sadda, Emma Venard, Tasneem Ekhtiar, Colin Powers, Sharon L. Darby, Ping Zheng, Shahin Ayazi

**Affiliations:** a Foregut Division, Department of Surgery, Allegheny Health Network, Pittsburgh, PA; b Chevalier Jackson Esophageal Research Center, Western Pennsylvania Hospital, Allegheny Health Network, Pittsburgh, PA; c Division of Abdominal Transplantation, Department of Surgery, University of Pittsburgh, Pittsburgh, PA; d School of Pharmacy, Duquesne University, Pittsburgh, PA; e Department of Surgery, Drexel University, Philadelphia, PA.

**Keywords:** adverse drug effects, dialysis, graft failure, graft function, kidney transplantation, proton pump inhibitors

## Abstract

This study evaluates the impact of proton pump inhibitor (PPI) use on graft-related outcomes following renal transplant. Proton pump inhibitors (PPIs) are frequently continued after kidney transplantation, often without a clear ongoing indication. Although their use has been associated with adverse renal outcomes in non-transplant populations, their clinical relevance in transplant recipients remains uncertain. We performed a retrospective cohort study using the TriNetX database of US health networks. We included 23,306 patients from 2000–2021 enrolled 4–6 weeks following their transplant. Patients were stratified by post-transplant PPI use and propensity score matched. The primary outcome was graft survival over 5 years; secondary outcomes included need for dialysis, renal function, and re-transplantation. Among 23,306 recipients, 15,601 patients (66.9%) were on PPI therapy 1 month post-transplant; 7553 matched pairs were analyzed. Post-transplant PPI use was associated with increased graft failure within 1 year (16.8% vs 9.4%; HR 1.79 [1.59–2.01]; *P* < .001) and between 1 and 5 years (12.8% vs 4.7%; HR 2.63 [2.27–3.05]; *P* < .001). Survival curves demonstrated early and sustained separation through 5 years (72.6% vs 85.8%; log-rank test *P* < .001). PPI exposure was also associated with increased dialysis dependence, progressive decline in graft function, and repeat transplantation, with greater risk observed with prolonged use (all *P* < .001). Post-transplant PPI use was common among kidney transplant recipients and was associated with a significantly increased risk of graft failure and worse kidney transplant outcomes. Persistent PPI use after kidney transplantation may represent a modifiable aspect of care, supporting periodic reassessment of acid suppression in transplant recipients.

## Graphical abstract

**Figure:**
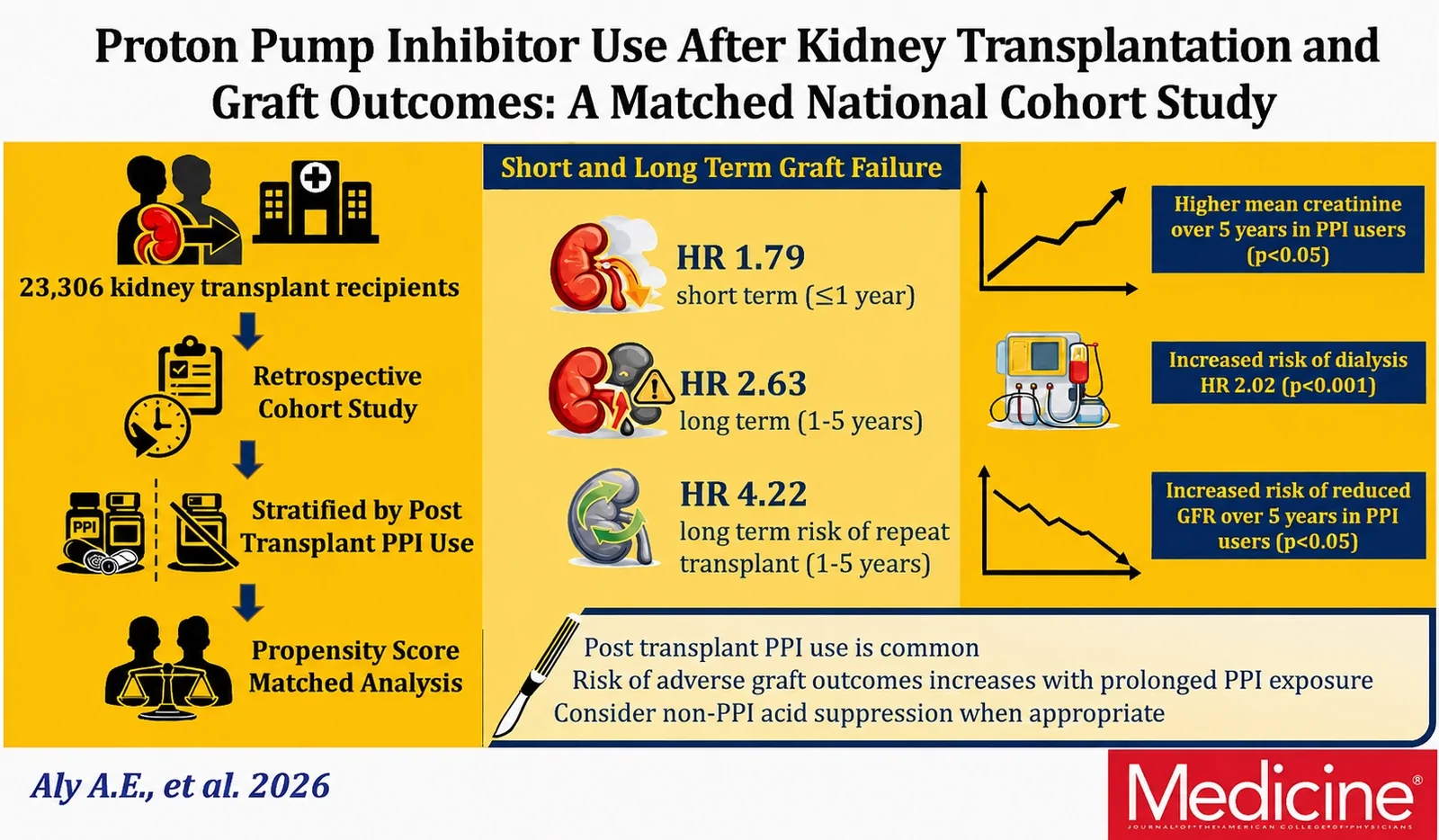

## 1. Introduction

Proton pump inhibitors (PPIs) are among the most frequently prescribed medications in the United States, with nearly a twofold increase in use over the past 2 decades.^[1,2]^ They are first-line therapy for acid-related gastrointestinal disorders, including gastroesophageal reflux disease (GERD), peptic ulcer disease, Barrett’s esophagus, and *Helicobacter pylori* treatment. Increasing evidence has linked long-term PPI exposure to adverse renal outcomes, including acute interstitial nephritis, hypomagnesemia, progression of chronic kidney disease (CKD), and increased risk of end-stage kidney disease (ESRD) in the general population.^[3-5]^

Kidney transplant recipients represent a uniquely vulnerable population, making these concerns highly relevant given their baseline renal impairment and susceptibility to graft dysfunction and failure. Prior studies of PPI use patterns in transplant recipients have shown that more than half of patients receiving PPIs before transplantation remain on therapy 1 year postoperatively, often without a clearly documented indication.^[6]^ Despite growing evidence of PPI-associated renal harm, data regarding their impact on kidney transplant outcomes remain conflicting. Some studies have demonstrated associations between PPI use and reduced graft function or increased mortality, whereas others have not found a significant relationship.^[7-9]^

In this study, we used a large national database to evaluate the association between PPI use and graft-related outcomes in kidney transplant recipients. Given the high prevalence of PPI exposure in this population and conflicting prior evidence, we sought to assess short- and long-term graft outcomes, renal function, and survival among transplant recipients exposed to PPIs. Knowing the adverse renal effects of PPIs in the general population, we believe transplant recipients on PPI therapy are likely to demonstrate worse outcomes given their baseline compromised renal reserve.

## 2. Materials and methods

### 2.1. Data source

This is a retrospective cohort study assessing the impact of proton pump inhibitor (PPI) use on kidney transplant outcomes. This study used patient data from the TriNetX Live (TriNetX LLC, Cambridge, MA) platform, a multicenter federated electronic network that aggregates de-identified electronic medical record (EMR) data from over 100 healthcare systems in the United States, encompassing millions of patients. The network provides comprehensive longitudinal patient data, including demographics, diagnoses (International Classification of Diseases, 10th Revision [ICD-10] codes), procedures (current procedural terminology [CPT] codes), medications, laboratory results, and clinical encounters. Data quality assurance occurs at the time of extraction, and all patient information is de-identified in compliance with the Health Insurance Portability and Accountability Act (HIPAA) Privacy Rule, with verification by an independent expert. Since the data used in this study were completely de-identified, institutional review board approval was not necessary, and informed consent from participants was waived.

### 2.2. Study population and cohort definition

Adult patients aged 18 years or older who underwent kidney transplantation between 2000 and 2021 were identified using CPT codes 50360 and 50365. Patients were enrolled between 4 and 6 weeks following their transplant, then subsequently stratified by PPI use, identified using RxNorm codes, beyond the immediate postoperative period defined as 1 month following transplant. The PPI cohort included recipients with documented PPI use beyond the defined postoperative period, whereas the control cohort consisted of recipients without any PPI use. Codes used for cohort identification are detailed in Table S1, Supplemental Digital Content 1.

### 2.3. Study outcomes

Outcomes were assessed within 5 years following transplantation and categorized as early (≤1 year) and late (1–5 years). The latest follow-up information available in the database was used for patients. The primary outcome assessed was kidney transplant failure, defined as ICD-10 code T86.12. Graft failure is considered death-censored, as post-transplant patients who died during the study period without experiencing graft failure or receiving the specific diagnostic code were not considered in the primary study outcome. Secondary outcomes included the need for dialysis, dialysis access creation or revision, repeat transplantation, and all-cause mortality. For graft function analyses, the last available estimated glomerular filtration rate (eGFR) value within each prespecified time interval (1, 3, and 5 years after transplantation) was used to categorize patients into clinically relevant eGFR thresholds using Kidney Disease: Improving Global Outcomes (KDIGO) CKD staging in transplant recipients. Patients with CKD stage 4T (CKD4T), representing severely reduced graft function, were defined as having an eGFR of 15 to 30 mL/min/1.73 m^2^, while CKD stage 5T (CKD5T), representing advanced graft dysfunction, was defined as an eGFR < 15 mL/min/1.73 m^2^. Additionally, the last available creatinine values within prespecified time intervals were used to estimate the mean creatinine at given time points following transplant. Subgroup analyses were performed to compare outcomes across prespecified cohorts, including recipients stratified by age (<60 vs ≥60 years), patterns of pre- and post-transplant PPI use, recipients requiring repeat transplantation, and recipients undergoing simultaneous kidney–pancreas transplantation. Sensitivity analysis in recipients receiving histamine-2 receptor (H2 receptor) antagonists was performed. The CPT, ICD-10, RxNorm, and logical observation identifiers, names and codes (LOINC) used to define the outcomes and subgroup cohorts analyzed are provided in Table S1, Supplemental Digital Content 1.

### 2.4. Statistical analysis

One-to-one propensity score matching was performed to balance age, sex, race, body mass index (BMI), diabetes mellitus, cardiopulmonary disease, kidney graft function 1 month after transplant, and immunosuppressive medication. Propensity scores were generated using logistic regression, and matched cohorts were created using greedy nearest-neighbor matching with a caliper width of 0.1 pooled standard deviations. Donor type and donor-specific characteristics, as well as post-transplant rejection episodes and BK polyomavirus infection, were not included in the propensity model because these variables were not reliably available in the TriNetX dataset.

For unmatched and matched data, baseline characteristics were summarized using means with standard deviations for continuous variables, and frequency and percentage for categorical variables. The Student *t* test or Chi-square test was performed as appropriate. A 2-sided *P* value of <.05 was considered statistically significant.

For the matching data, the association between PPI use and a time-to-event outcome was estimated using a Cox proportional hazards regression model and presented by hazard ratio with 95% confidence interval (CI). A Kaplan–Meier survival plot was generated for a time-to-event outcome, along with the log-rank test assessing differences for 2 survival curves. A 2-sided *P*-value of <.05 or 95% CI for the hazard ratio that did not cross 1 indicated statistical significance.

All statistical analyses were performed using the TriNetX analytics platform, with results of survival analyses internally validated against SAS version 9.4.

## 3. Results

### 3.1. Study population

A total of 23,306 kidney transplant recipients were identified. The mean (SD) age at transplantation was 50.4 (14.9) years; 60.0% were male; and the mean follow-up was 53.9 (13.7) months. Overall, 15,601 patients (66.9%) received PPIs beyond the defined postoperative period. At baseline, PPI users had a higher prevalence of comorbidities, including diabetes, coronary artery disease, and chronic obstructive pulmonary disease. After propensity score matching, 2 cohorts of 7553 patients each were generated. Prevalence of reflux disease remained elevated in the PPI users, but kidney function at the time of patient enrollment post-transplant was similar between cohorts. Baseline demographics, clinical characteristics, kidney function, and immunosuppressive medication are highlighted in Table 1.

**Table 1:** Baseline demographics and clinical characteristics of post-transplant PPI users and non-users before and after propensity score matching.

Characteristics	Before matching			After matching		
	PPI users n = 15,601	No PPI use n = 7,705	*P*-value	PPI users n = 7,553	No PPI use n = 7,553	*P*-value
Age, mean (SD)	50.9 (14.6)	49.3 (15.5)	<.001	49.7 (15.2)	49.4 (15.5)	.278
Male, n (%)	9019 (57.8)	4969 (64.5)	<.001	4884 (64.7)	4930 (65.3)	.433
White, n (%)	8329 (53.4)	4288 (55.7)	<.001	4327 (57.3)	4269 (56.5)	.341
BMI, mean (SD)	27.4 (5.7)	27.0 (5.4)	<.001	27.1 (5.6)	27.0 (5.4)	.109
Comorbidities						
Diabetes, n (%)	7244 (46.4)	3012 (39.1)	<.001	3049 (40.4)	3009 (39.8)	.507
Hypertension, n (%)	12,179 (78.1)	6013 (78.0)	.310	5989 (79.3)	5977 (79.1)	.810
CAD, n (%)	3719 (23.8)	1355 (17.6)	<.001	1338 (17.7)	1353 (17.9)	.750
CVD, n (%)	621 (4.0)	244 (3.2)	.003	276 (3.7)	244 (3.2)	.153
COPD, n (%)	460 (2.9)	164 (2.1)	<.001	192 (2.5)	163 (2.2)	.119
GERD, n (%)	4827 (30.9)	1349 (17.5)	<.001	2346 (31.1)	1334 (17.7)	<.001
Kidney function						
GFR (mL/min/1.73m^2^), mean (SD)	52.0 (23.9)	54.9 (22.2)	<.001	54.2 (24.2)	54.8 (22.2)	.095
Immunosuppression use						
Tacrolimus, n (%)	14,160 (90.8)	6474 (84.0)	<.001	6464 (85.6)	6471 (85.7)	.871
Mycophenolate mofetil, n (%)	12,532 (80.3)	5853 (76.0)	<.001	5938 (78.6)	5844 (77.4)	.065
Mycophenolic acid, n (%)	5877 (37.7)	2626 (34.1)	<.001	2517 (33.3)	2621 (34.7)	.074
Prednisone, n (%)	11,859 (76.0)	5277 (68.5)	<.001	5350 (70.8)	5263 (69.7)	.121
Hydrocortisone, n (%)	3013 (19.3)	1330 (17.3)	<.001	1307 (17.3)	1329 (17.6)	.637

BMI = body mass index, CAD = coronary artery disease, COPD = chronic obstructive pulmonary disease, CVD = cerebrovascular disease, GERD = gastroesophageal reflux disease, GFR = glomerular filtration rate, PPI = proton pump inhibitor, SD = standard deviation.

### 3.2. Outcomes in PPI users before matching

Unmatched outcomes within 1 year and beyond 1 year after transplantation are summarized in Table S2, Supplemental Digital Content 2. Within the first year, graft failure occurred in 17.1% of PPI users compared with 9.1% of non-users (hazard ratio [HR] 1.93 [1.76–2.13], *P* < .001). PPI users also had higher rates of dialysis and dialysis access creation or revision. Repeat transplantation was uncommon in both groups but occurred more frequently among PPI users (0.6% vs 0.2%, HR 2.90 [1.65–5.10], *P* < .001). Beyond 1 year of follow-up, graft failure remained higher among PPI users (12.8% vs 4.4%, HR 2.82 [2.42–3.27], *P* < .001), as did rates of dialysis and access-related procedures. The rates of repeat transplantation remained low, but the difference became more pronounced over time (1.0% vs 0.2%, HR 4.23 [2.49–7.21], *P* < .001).

### 3.3. Outcomes in PPI users after matching

After propensity score matching, PPI use remained associated with worse outcomes (Table 2). In the first year after transplantation, graft failure occurred in 16.8% of PPI users compared with 9.4% of non-users (HR 1.79 [1.59–2.01], *P* < .001). PPI users continued to have higher rates of dialysis, dialysis access creation or revision, and repeat transplantation. Beyond 1 year, graft failure remained more frequent in the PPI cohort (12.8% vs 4.7%, HR 2.63 [2.27–3.05], *P* < .001), with a larger difference in repeat transplantation rates (1.1% vs 0.2%, HR 4.22 [2.53–7.03], *P* < .001).

**Table 2:** Primary and secondary outcomes in transplant recipients stratified by PPI use post transplantation after propensity score matching.

	PPI users	No PPI use	Hazard ratio (95% CI)	*P*-value
Early outcomes (≤1 yr)				
Kidney transplant failure, %	16.8%	9.4%	1.79 (1.59–2.01)	<.001
Need for dialysis, %	20.7%	12.1%	1.77 (1.62–1.93)	<.001
Dialysis access procedures, %	10.9%	4.3%	2.61 (2.27–3.01)	<.001
Re-transplant, %	0.6%	0.2%	3.04 (1.62–5.68)	<.001
Late outcomes (1–5 yr)				
Kidney transplant failure, %	12.8%	4.7%	2.63 (2.27–3.05)	<.001
Need for dialysis, %	22.1%	10.8%	2.02 (1.86–2.20)	<.001
Dialysis access procedures, %	14.8%	4.8%	3.05 (2.71–3.44)	<.001
Re-transplant, %	1.1%	0.2%	4.22 (2.53–7.03)	<.001

CI = confidence interval, PPI = proton pump inhibitor.

Kaplan–Meier analysis demonstrated early and persistent separation in graft survival, with significantly lower survival among PPI users over follow-up (log-rank test: *P* < .001) (Fig. 1).

**Figure 1.**
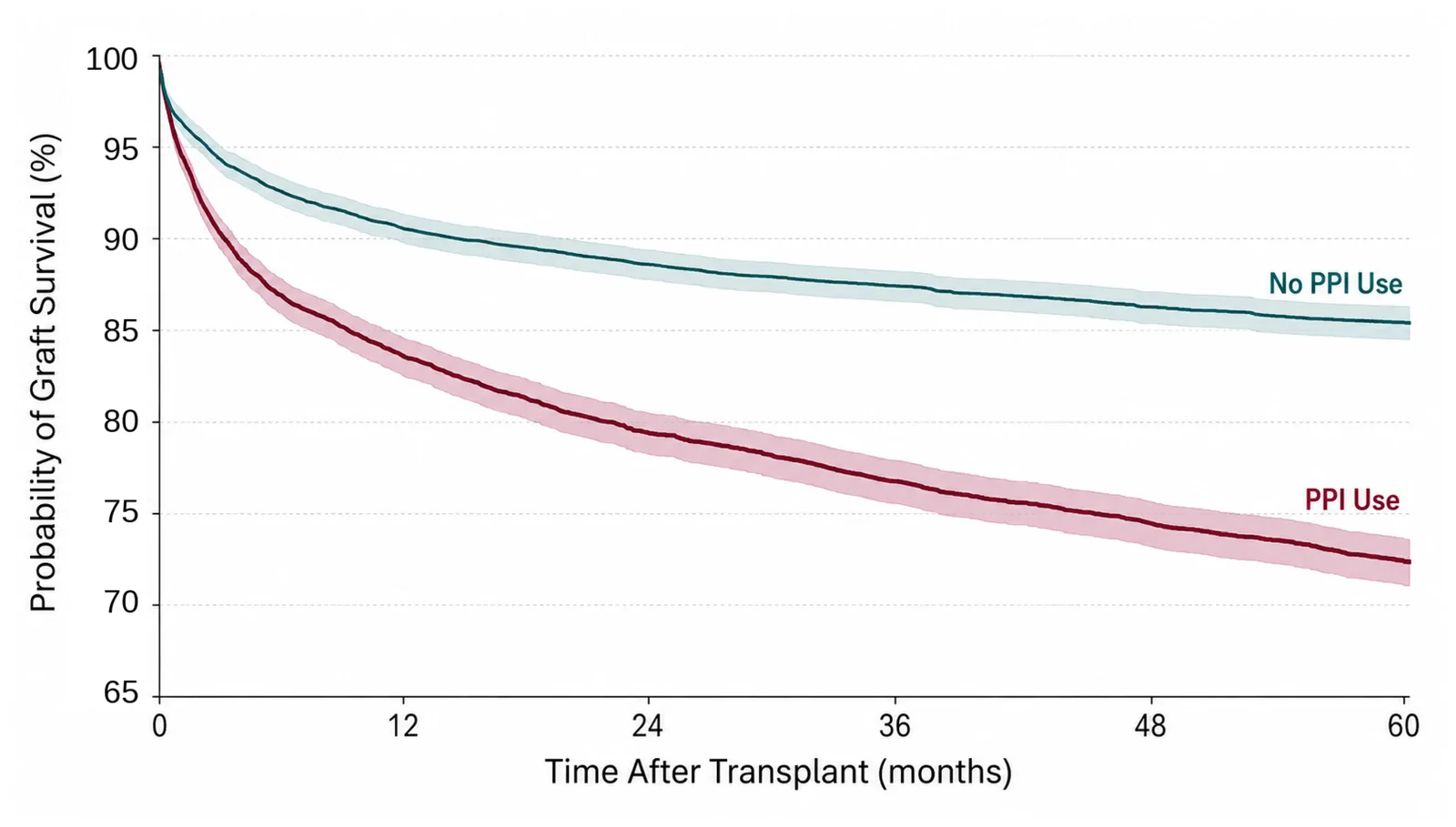
Kaplan–Meier curve demonstrating kidney graft survival in PPI users versus non-PPI users. PPI users experienced significantly lower graft survival compared with non-users throughout the 60-month follow-up period. Estimated graft survival at 60 months for PPI users was 72.6% compared to 85.8% in the non-PPI users (log-rank, *P* < .001). Shaded areas represent 95% confidence intervals. PPI = proton pump inhibitor.

### 3.4. Association between PPI use and graft function

The risk of severely reduced graft function (CKD4T; eGFR 15–30 mL/min/1.73 m^2^) and advanced graft dysfunction (CKD5T; eGFR < 15 mL/min/1.73 m^2^) across different follow-up intervals is illustrated in Figure 2. Over 1, 3, and 5-year follow-up, PPI users had higher odds of severely reduced graft function (CKD4T; odds ratio [OR] 1.74 [95% CI, 1.61–1.88], OR 2.09 [95% CI, 1.94–2.25], and OR 2.32 [95% CI, 2.13–2.54], respectively; all *P* < .001). The corresponding odds of advanced graft dysfunction (CKD5T) were also higher among PPI users (OR 2.26 [95% CI, 1.95–2.63], OR 2.54 [95% CI, 2.22–2.91], and OR 2.67 [95% CI, 2.38–3.00], respectively; all *P* < .001). Consistent with these findings, longitudinal analysis demonstrated persistently higher mean serum creatinine levels among PPI users throughout follow-up, with an increasing separation between groups over time (Fig. 3).

**Figure 2.**
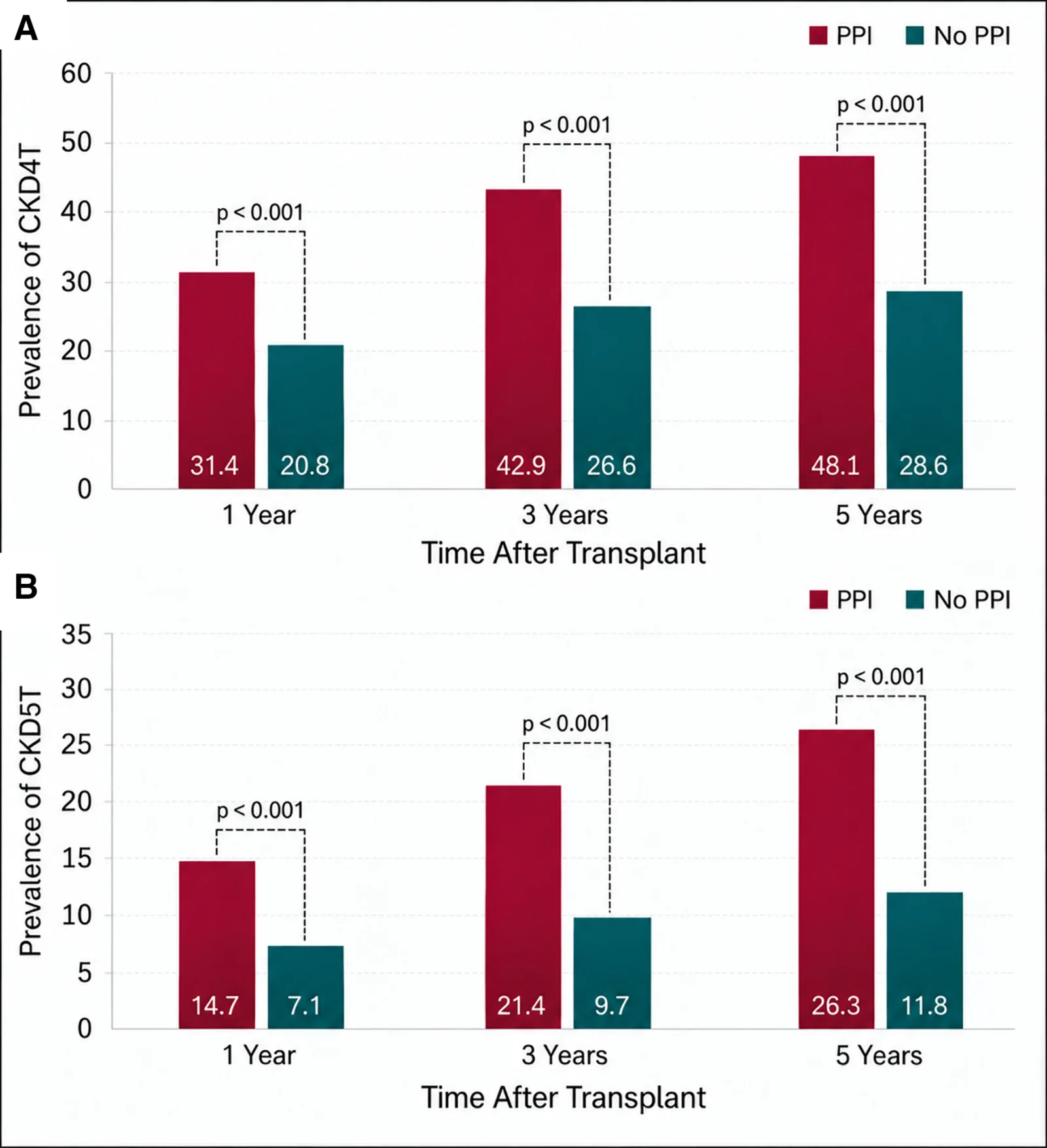
Prevalence of reduced graft function across post-transplant follow-up intervals stratified by PPI use. (A) The prevalence of CKD4T, representing severely reduced graft function (eGFR 15–30 mL/min/1.73m^2^) was significantly higher in the PPI use cohort over 1, 3, and 5-year post-transplant follow-up intervals (all *P* < .001). (B) The prevalence of CKD5T, representing advanced graft dysfunction (eGFR < 15 mL/min/1.73m^2^) was similarly higher in PPI users across the follow-up intervals (all *P* < .001). CKD = chronic kidney disease, eGFR = estimated glomerular filtration rate, PPI = proton pump inhibitor.

**Figure 3.**
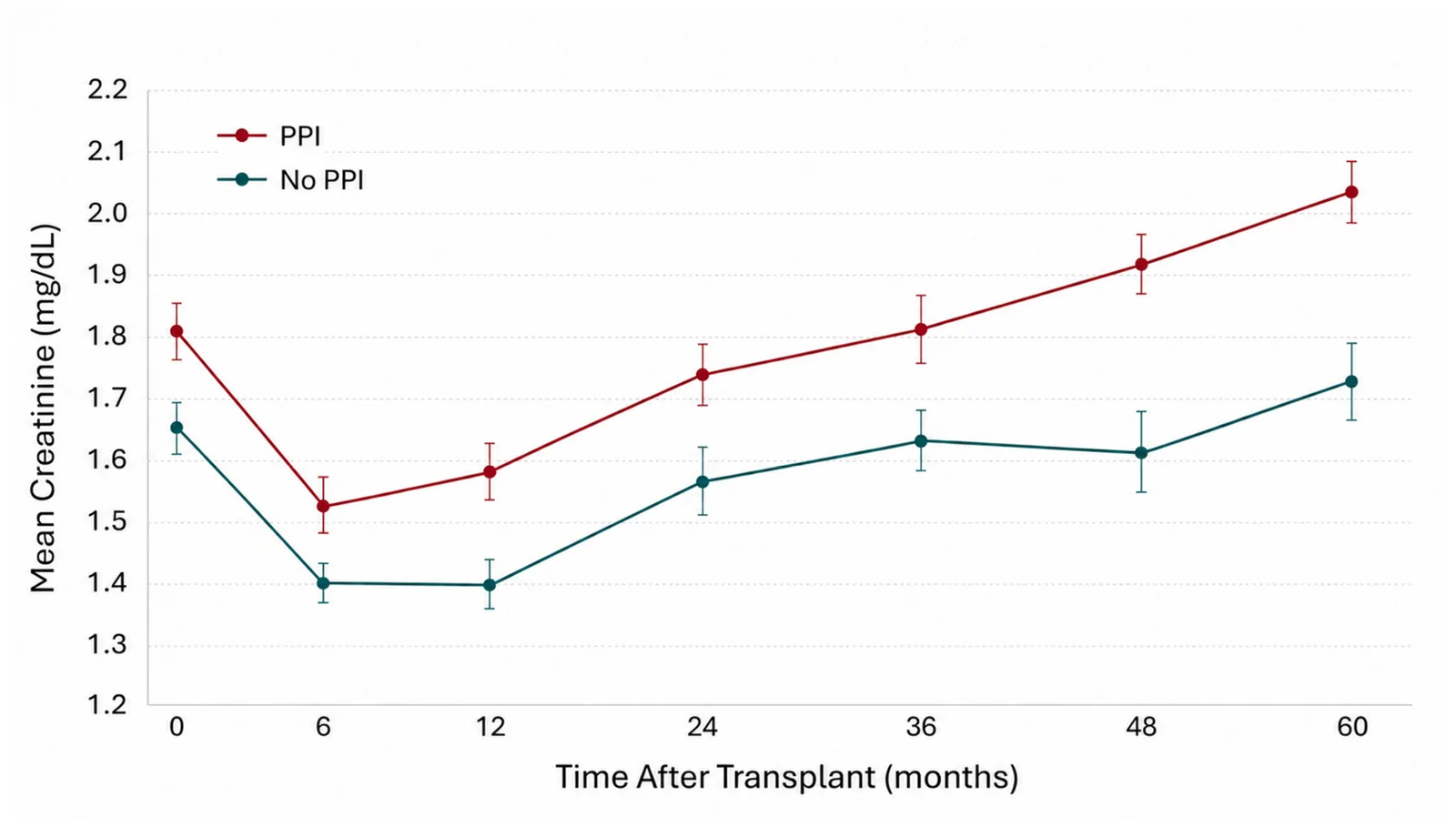
Mean serum creatinine over time in PPI users versus non-users following kidney transplantation. PPI users demonstrated consistently higher creatinine levels over the follow-up period. There is a clear and persistent separation between the 2 cohorts, with the difference increasing with time. At all follow-up points, comparisons of mean creatinine were statistically significant (*P* < .05). Error bars show standard error. PPI = proton pump inhibitor.

### 3.5. All-cause mortality

All-cause mortality was higher among PPI users at the end of follow-up (HR 1.36 [1.19–1.55], *P* < .001). After propensity score matching, 5-year survival remained lower in PPI users compared with non-users (88.3% vs 91.3%; log-rank test *P* < .001).

### 3.6. Age-stratified outcomes

PPI use was associated with higher graft failure rates within 1 year and between 1 and 5 years after transplantation in both recipients younger than 60 years and those aged 60 years or older (all *P* < .001). Five-year graft survival was consistently lower among PPI users across age groups (<60 years: 72.4% vs 86.3%; ≥60 years: 73.6% vs 89.5%; both log-rank test *P* < .001).

### 3.7. Pre-transplant PPI exposure and post-transplant continuation

Among post-transplant PPI users, 27.7% received PPIs within the year prior to transplantation. Pre-transplant PPI exposure alone was not associated with graft failure. No difference in outcomes was observed in patients on PPI therapy after transplant, regardless of preoperative use (*P* > .05). In contrast, in recipients with pre-transplant PPI exposure, continuation of PPIs after transplant was associated with higher graft failure both within 1 year (14.4% vs 8.1%, HR 1.72 [1.14–2.60], *P* = .009) and between 1 and 5 years (12.2% vs 4.1%, HR 2.72 [1.52–4.84], *P* < .001) compared with discontinuation.

### 3.8. Duration of post-transplant PPI use

Among post-transplant PPI users, 7868 patients (50.4%) remained on therapy beyond 1 year. Compared with recipients who used PPIs for ≤ 1 year, no difference in graft failure was seen within 1 year of transplant (18.5% vs 18.1%, HR 1.00 [0.85–1.17], *P* = .962). However, there was a significant increase in the risk of graft failure seen between 1 and 5 years in patients who persisted on PPIs compared to those who stopped (14.2% vs 5.2%, HR 2.35 [1.79–3.09], *P* < .001).

### 3.9. Comparison between PPIs and H2 blockers

Compared with recipients receiving H2 blockers, PPI users had higher rates of graft failure within 1 year (14.9% vs 12.1%, HR 1.24 [1.06–1.45], *P* = .009) and between 1 and 5 years after transplantation (10.2% vs 6.3%, HR 1.59 [1.28–1.98], *P* < .001).

### 3.10. Repeat transplant outcomes

Among 1068 recipients who underwent repeat kidney transplantation, those who continued PPIs after re-transplantation (n = 855) had higher rates of repeat graft failure over a mean follow-up of 45.9 (19.6) months (35.4% vs 25.4%, *P* = .009). Repeat graft survival was lower among persistent PPI users (60.9% vs 71.5%, log-rank test *P* = .008).

### 3.11. Outcomes in multi-organ transplant recipients

In 1147 recipients of simultaneous kidney–pancreas transplantation, post-transplant PPI use was associated with higher rates of graft failure over 5 years at a mean follow-up of 56.0 (11.6) months (33.9% vs 19.5%, HR 1.80 [1.09–2.98], log-rank test *P* = .021).

## 4. Discussion

Proton pump inhibitors are frequently prescribed to kidney transplant recipients, either continued from pre-transplant regimens or newly initiated after transplantation, and are often used for prolonged durations without a clearly documented indication.^[6,10]^ Prior studies have reported associations between PPI use and adverse outcomes in this population, including antibody-mediated rejection, reduced quality of life, and increased mortality.^[7,8,11]^ However, existing data remain inconsistent, and evidence specifically examining graft failure has been limited by small sample sizes, heterogeneous follow-up, and single-center designs. In this large national cohort of kidney transplant recipients, we evaluated the association between post-transplant PPI use and graft-related outcomes. PPI use was associated with higher rates of graft failure, dialysis requirement and access procedures, repeat transplantation, and decreased graft function, with effects observed both within the first year and over longer-term follow-up. These findings extend prior literature by providing robust, population-level evidence linking persistent post-transplant PPI use with adverse graft-related outcomes.

In this cohort of over 23,000 kidney transplant recipients, the majority were maintained on PPI therapy after transplantation (66.9%), consistent with prior reports describing high post-transplant PPI utilization.^[7,11]^ Over 5 years of follow-up, PPI use was associated with adverse kidney transplant outcomes, including higher rates of graft failure observed both early and over longer-term follow-up. Although PPI users had a greater burden of comorbidities at baseline, including diabetes, coronary artery disease, and chronic obstructive pulmonary disease, these differences did not fully explain the observed outcomes. After propensity score matching, PPI use remained associated with significantly higher rates of early graft failure (16.8% vs 9.4%) and graft loss between 1 and 5 years (12.8% vs 4.7%). In addition, PPI users demonstrated higher rates of dialysis, dialysis access procedures, and repeat transplantation, reinforcing the consistency of the association across multiple graft-related endpoints.

Our findings are consistent with accumulating evidence linking prolonged PPI exposure to renal injury, particularly in kidney transplant recipients. Beyond the first post-transplant year, chronic rejection, most commonly antibody-mediated rejection, accounts for most graft failures, reported in up to 63% of cases.^[12,13]^ Several studies have suggested a potential association between PPI use and antibody-mediated rejection. In a meta-analysis of 4844 kidney transplant recipients, PPI use was associated with a higher risk of antibody-mediated rejection compared with H2 blocker therapy (OR 1.87, 95% CI, 1.03–3.04).^[8]^ However, this relationship remains debated, as smaller single-center retrospective studies have not demonstrated a significant association between PPI use and rejection rates.^[9,11]^

Beyond graft-related outcomes, we also observed a significant difference in patient survival. Five-year all-cause mortality was slightly higher among PPI users, with survival analysis showing 88.3% versus 91.3% survival at the end of the study follow-up (log rank *P* < .001). This aligns with similar studies reporting higher mortality among transplant recipients on long-term PPIs, with increased risk seen in patients on high-dose PPIs.^[7]^ Our Kaplan–Meier analysis further demonstrated this finding, showing early divergence in graft survival that widened throughout the follow-up period, indicating a persistent association between PPI use and lower graft survival.

The effect of PPI use on kidney graft function remains incompletely defined. In our cohort, PPI use was associated with a significant and progressive decline in graft function, as measured by estimated GFR. Compared with non-users, PPI users had a modest increase in risk of severely decreased graft function and being classified as CKD4T (eGFR 15–30 mL/min/1.73 m^2^), which accumulated over time (OR 1.74 at 1 year, 2.09 at 3 years, and 2.32 at 5 years). The association was even stronger for advanced graft dysfunction (CKD5T; eGFR < 15 mL/min/1.73 m^2^), with consistently elevated odds across all follow-up intervals (OR 2.26, 2.54, and 2.67 at 1, 3, and 5 years, respectively). These findings suggest a sustained association between PPI exposure and declining graft function from early to late post-transplant periods. Prior studies evaluating the relationship between PPI use and graft function have yielded mixed results. Flothow et al reported a marginal association between higher PPI dose and a >30% decline in eGFR between 6 months and 2 years post-transplant but did not identify consistent associations with further renal decline.^[10]^ Similarly, a meta-analysis of 14 observational studies found no significant association between PPI use and changes in renal function within the first year after transplantation, a finding the authors noted was discordant with evidence from the general population.^[14]^ Importantly, the eGFR thresholds examined in our study are clinically meaningful in transplant recipients. Prior work by Kasiske et al demonstrated that post-transplant eGFR < 30 and < 15 mL/min/1.73 m^2^ are strongly associated with subsequent graft loss (HR 2.86 for eGFR 15–30 and HR 13.2 for eGFR < 15), underscoring the clinical relevance of the functional decline observed among PPI users in our cohort.^[15-17]^

Donor quality is a major determinant of post-transplant graft survival, with higher Kidney Donor Profile Index (KDPI) scores consistently associated with delayed graft function, graft dysfunction, and long-term graft loss.^[18,19]^ The TriNetX dataset did not provide living-versus-deceased donor status or the donor-level variables required to calculate KDPI; thus, donor type and donor quality could not be directly adjusted for in the propensity model. As a pragmatic approach, we performed age-stratified analyses, using recipient age as a surrogate for donor quality, given that younger recipients are more likely to receive lower-KDPI organs.^[18]^ Across both age groups (<60 and ≥60 years), PPI use remained associated with higher rates of graft failure and worse graft survival, with comparable risk observed in both early and later post-transplant periods. Although recipient age is an imperfect proxy for donor quality, the consistency of these findings across age strata suggests that the observed association between PPI use and adverse graft outcomes is unlikely to be explained solely by differences in donor quality. Future studies incorporating granular donor-level data are needed to further clarify the interaction between PPI exposure and donor graft characteristics.

Nearly one-third of kidney transplant recipients in this cohort had documented PPI use in the year preceding transplantation. Among these patients, continued PPI use after transplantation was associated with significantly higher rates of graft failure compared with discontinuation, both within the first year (14.4% vs 8.1%) and between 1 and 5 years (12.2% vs 4.1%). In contrast, among post-transplant PPI users, pretransplant PPI exposure alone was not associated with graft failure, suggesting that prior exposure did not independently influence post-transplant outcomes.

We further examined the impact of the duration of post-transplant PPI exposure. Within the first year, with PPI use being similar, outcomes were comparable with no significant difference in graft failure seen (HR 1.00, *P* = .962). However, compared with the recipients who discontinued PPIs within one year after transplantation, prolonged PPI use was associated with a substantially higher risk of graft failure during longer-term follow-up (HR 2.35, *P* < .001). The association observed with prolonged PPI use is consistent with population-based studies demonstrating cumulative renal risk with long-term PPI exposure, including progression of chronic kidney disease and end-stage renal disease.^[4,5,20]^ Proposed mechanisms include acute interstitial nephritis with persistent tubulointerstitial injury, hypomagnesemia, vascular injury, and endothelial dysfunction, all of which may worsen with sustained exposure.^[21,22]^ Collectively, these findings support the importance of duration of post-transplant PPI exposure as a key factor associated with progressive graft dysfunction.

The observed association between PPI use and adverse graft outcomes may be partially mediated through interactions with immunosuppressive therapy. Standard post–kidney transplant immunosuppression typically includes a calcineurin inhibitor (most commonly tacrolimus), an antiproliferative agent such as mycophenolate mofetil, and corticosteroids. Mycophenolate mofetil is a prodrug that undergoes gastric hydrolysis, and alterations in gastric pH have been shown to affect its absorption and bioavailability.^[23,24]^ Acid suppression may therefore reduce effective exposure to mycophenolate, potentially increasing susceptibility to rejection and graft-related complications.

To explore whether the associations observed in our study were specific to PPI therapy rather than acid suppression more broadly, we compared outcomes between recipients receiving PPIs and those treated with H2 receptor antagonists. PPI use remained associated with higher rates of graft failure, with the difference more pronounced beyond 1 year after transplantation. Compared with H2 blocker users, PPI users had statistically significantly higher rates of graft failure both within the first year (14.9% vs 12.1%) and between 1 and 5 years after transplant (10.2% vs 6.3%). The emergence of potassium-competitive acid blockers (PCABs) provides an opportunity for future studies to evaluate their safety and potential impact on graft-related outcomes in kidney transplant recipients.

While our study demonstrated an association between PPI use and increased renal transplant graft failure in a large national cohort, there remain several limitations inherent to large administrative databases. The database used for analysis did not include donor-specific variables, limiting adjustment for factors known to influence graft outcomes. Differentiation between living donor and deceased donor grafts was not available for analysis. Certain baseline characteristics known to impact graft survival, such as BK virus infection, were not included in baseline propensity matching between cohorts as no ICD-10 code specifically identifies history of infection. Additionally, given the database’s limitation, characteristics required to calculate the Kidney Donor Profile Index, including donor age, terminal creatinine, race, hepatitis C virus status, cause of death, and cold ischemia time, were therefore unavailable for matching or risk adjustment. To partially address this limitation, we performed age-stratified analyses using recipient age as a surrogate for donor quality, based on established allocation patterns in which younger recipients more frequently receive lower-KDPI organs. The persistence of worse graft outcomes among PPI users across both age strata suggests that the observed association is unlikely to be explained solely by differences in donor quality. Additionally, granular data on immunosuppressive regimens, including dosage and regimen changes at the patient level, were not available for analysis. Future studies using UNOS registry data or institutional transplant datasets with comprehensive donor and recipient variables are needed to further clarify the independent impact of PPI exposure on graft-related outcomes. Additionally, given the nature of the database, dosing information and exact timing of initiation of medications are not available, which limits conclusions regarding the interactions of PPIs and immunosuppressive regimens.

## 5. Conclusion

In this large retrospective cohort of kidney transplant recipients, post-transplant PPI use was consistently associated with higher rates of graft failure, dialysis and dialysis-access procedures, repeat transplantation, and impaired graft function. These associations remained robust across multiple sensitivity analyses, including age-stratified cohorts used as a surrogate for donor quality and comparisons with alternative acid-suppressive therapies. The consistency of these findings across clinical contexts suggests a meaningful association between PPI exposure and adverse graft outcomes after transplantation. These results underscore the importance of careful indication and periodic reassessment of acid-suppressive therapy in kidney transplant recipients. Future studies incorporating detailed donor characteristics and medication exposure data are needed to better define underlying mechanisms and to determine whether reducing unnecessary PPI use may improve long-term graft outcomes.

## Author contributions

**Conceptualization:** Ahmed E. Aly, Vineeth R. Sadda, Colin Powers, Shahin Ayazi.

**Data curation:** Ahmed E. Aly, Emma Venard, Tasneem Ekhtiar, Ping Zheng.

**Formal analysis:** Ahmed E. Aly, Sharon L. Darby, Ping Zheng.

**Investigation:** Tasneem Ekhtiar.

**Methodology:** Ahmed E. Aly, Vineeth R. Sadda, Emma Venard, Colin Powers, Sharon L. Darby, Ping Zheng, Shahin Ayazi.

**Supervision:** Shahin Ayazi.

**Writing – original draft:** Ahmed E. Aly.

**Writing – review & editing:** Vineeth R. Sadda, Emma Venard, Tasneem Ekhtiar, Colin Powers, Sharon L. Darby, Ping Zheng, Shahin Ayazi.




