# Supplementary material for: Proton pump inhibitor use after kidney transplantation and graft outcomes: A matched national cohort study
Source: Medicine (Baltimore). 2026 Oct 9;105(41):e51046. doi: 10.1097/MD.0000000000051046 (PMC13657520; doi:10.1097/MD.0000000000051046)
Supplement: Supplementary file 1 [file medi-105-e51046-s001.docx]

| Supplementary Table 1: ICD-10, CPT, RxNorm, and LOINC Codes Used | | | |
| --- | --- | --- | --- |
| Term | ICD-10, CPT, RxNorm, or LOINC Description | ICD-10, CPT, RxNorm, or LOINC | Codes |
| Renal Transplant | Renal allotransplantation, implantation of graft; without recipient nephrectomy | CPT | 50360 |
|  | Renal allotransplantation, implantation of graft; with recipient nephrectomy |  | 50365 |
| Pancreas Transplant | Transplantation of pancreatic allograft |  | 48554 |
| PPI | Pantoprazole | RxNorm | 40790 |
|  | Omeprazole |  | 7646 |
|  | Esomeprazole |  | 283742 |
|  | Rabeprazole |  | 114979 |
|  | Lansoprazole |  | 17128 |
|  | Dexlansoprazole |  | 816346 |
| Transplant Failure | Kidney transplant failure | ICD-10 | T86.12 |
| GERD | Gastroesophageal refux disease | ICD-10 | K21 |
| H2 Blockers | Famotidine | RxNorm | 4278 |
|  | Cimetidine |  | 2541 |
|  | Nizatidine |  | 42319 |
| Dialysis Procedures | Dependence on renal dialysis | ICD-10 | Z99.2 |
|  | Dialysis services and procedures | CPT | 1012740 |
|  | Hemodialysis procedures |  | 1012752 |
|  | Hemodialysis procedure with single evaluation by a physician or other qualified health care professional |  | 90935 |
|  | Dialysis procedure other than hemodialysis (eg, peritoneal dialysis, hemofiltration, or other continuous renal replacement therapies), with single evaluation by a physician or other qualified health care professional |  | 90947 |
|  | Dialysis procedure other than hemodialysis (eg, peritoneal dialysis, hemofiltration, or other continuous renal replacement therapies) requiring repeated evaluations by a physician or other qualified health care professional, with or without substantial revision of dialysis prescription |  | 90945 |
|  | Hemodialysis procedure requiring repeated evaluation(s) with or without substantial revision of dialysis prescription |  | 90937 |
| Dialysis Access Creation or Revision | Dialysis circuit procedures | CPT | 1029674 |
|  | Insertion of cannula for hemodialysis, other purpose |  | 1006748 |
|  | Arteriovenous anastomosis, open |  | 1006752 |
|  | Creation of arteriovenous fistula by other than direct arteriovenous anastomosis |  | 1006759 |
|  | Revision, open, arteriovenous fistula |  | 1006763 |
|  | External cannula declotting (separate procedure) |  | 1006769 |
|  | Thrombectomy, open, arteriovenous fistula without revision, autogenous or nonautogenous dialysis graft |  | 36831 |
|  | Percutaneous arteriovenous fistula creation, upper extremity, single access of both the peripheral artery and peripheral vein, including fistula maturation procedures (eg, transluminal balloon angioplasty, coil embolization) when performed, including all vascular access, imaging guidance and radiologic supervision and interpretation |  | 36836 |
|  | Percutaneous arteriovenous fistula creation, upper extremity, separate access sites of the peripheral artery and peripheral vein, including fistula maturation procedures (eg, transluminal balloon angioplasty, coil embolization) when performed, including all vascular access, imaging guidance and radiologic supervision and interpretation |  | 36837 |
|  | Distal revascularization and interval ligation (DRIL), upper extremity hemodialysis access (steal syndrome) |  | 36838 |
|  | Thrombectomy, percutaneous, arteriovenous fistula, autogenous or nonautogenous graft (includes mechanical thrombus extraction and intra-graft thrombolysis) |  | 36870 |
|  | Insertion of non-tunneled centrally inserted central venous catheter; age 5 years or older |  | 36556 |
|  | Insertion of tunneled intraperitoneal catheter for dialysis, open |  | 49421 |
|  | Insertion of tunneled centrally inserted central venous catheter, without subcutaneous port or pump; age 5 years or older |  | 36558 |
|  | Laparoscopy, surgical; with insertion of tunneled intraperitoneal catheter |  | 49324 |
| GFR | Glomerular filtration rate [Volume Rate/Area] in Serum or Plasma by Creatinine-based formula (MDRD)/1.73 sq M | LOINC | 33914-3 |
|  | Glomerular filtration rate [Volume Rate/Area] in Serum, Plasma or Blood by Creatinine-based formula (MDRD)/1.73 sq M among non black population |  | 48642-3 |
|  | Glomerular filtration rate [Volume Rate/Area] in Serum, Plasma or Blood by Creatinine-based formula (MDRD)/1.73 sq M among black population |  | 48643-1 |
|  | Glomerular filtration rate [Volume Rate/Area] in Serum, Plasma or Blood by based on 1.73 sq M |  | 69405-9 |
|  | Glomerular filtration rate/1.73 sq M.predicted by Creatinine-based formula (MDRD) in Blood |  | 76633-7 |
|  | Glomerular filtration rate [Volume Rate/Area] in Serum, Plasma or Blood by Creatinine-based formula (MDRD)/1.73 sq M |  | 77147-7 |
| Creatinine | Creatinine [Mass/volume] in Serum or Plasma | LOINC | 2160-0 |
|  | Creatinine [Mass/volume] in Blood |  | 38483-4 |
