# Supplementary material for: Proton pump inhibitor use after kidney transplantation and graft outcomes: A matched national cohort study
Source: Medicine (Baltimore). 2026 Oct 9;105(41):e51046. doi: 10.1097/MD.0000000000051046 (PMC13657520; doi:10.1097/MD.0000000000051046)
Supplement: Supplementary file 2 [file medi-105-e51046-s002.docx]

| Supplemental Table 2: Primary and Secondary Outcomes in Transplant Recipients Stratified by PPI Use Post Transplantation Without Propensity Score Matching | | | | |
| --- | --- | --- | --- | --- |
|  | PPI use | No PPI use | Hazards Ratio (95% CI) | p-value |
| **Early Outcomes (≤1year)** | | | | |
| Kidney transplant failure, % | 17.1% | 9.1% | 1.93 (1.76-2.13) | <0.001 |
| Need for dialysis, % | 20.5% | 12.9% | 1.64 (1.52-1.76) | <0.001 |
| Dialysis access procedures, % | 11.4% | 4.4% | 2.61 (2.32-2.94) | <0.001 |
| Re-Transplant, % | 0.6% | 0.2% | 2.90 (1.65-5.10) | <0.001 |
| **Late Outcomes (1-5 years)** | | | | |
| Kidney transplant failure, % | 12.8% | 4.4% | 2.82 (2.42-3.27) | <0.001 |
| Need for dialysis, % | 21.9% | 9.8% | 2.25 (2.07-2.46) | <0.001 |
| Dialysis access procedures, % | 14.2% | 4.5% | 3.11 (2.75-3.51) | <0.001 |
| Re-Transplant, % | 1.0% | 0.2% | 4.23 (2.49-7.21) | <0.001 |
